# Therapeutic potential of targeting *MYCN*: A case series report of neuroblastoma with *MYCN* amplification

**DOI:** 10.1097/MD.0000000000020853

**Published:** 2020-06-19

**Authors:** Can Huang, Shayi Jiang, Jingwei Yang, Xuelian Liao, Yanhua Li, Shanshan Li

**Affiliations:** Department of Hematology and Oncology, Children's Hospital of Shanghai, Shanghai Jiao Tong University, Shanghai, China.

**Keywords:** abdomen mass, *MYCN*, neuroblastoma, pediatric, targeting

## Abstract

**Introduction:**

Neuroblastoma (NB) with *MYCN* amplification has a poor prognosis and high mortality. The potential molecular biological relationship between clinical features and *MYCN* amplification should be explored.

**Methods:**

NB patients were examined by fluorescence in situ hybridization (FISH) for *MYCN* amplification in the tumor mass or bone marrow samples to determine whether *MYCN* was amplified. A series of eleven *MYCN*-amplified NB patients were included. The age, primary site, tumor size, specific biomarkers, and invaded organs were analyzed. All patients accepted standardized treatment of surgery, chemotherapy, and radiotherapy. Progression-free survival (PFS) and overall survival (OS) were evaluated.

**Results:**

The median age at diagnosis was 24 months. Nine patients (81.8%) were in stage IV, with high serum neuron-specific enolase (NSE) expression, normal urine vanillylmandelic acid (VMA) level and extensive metastases. All patients accepted a chemotherapy protocol with 8 to 10 cycles, and 9 patients (81.8%) were sensitive to the initial chemotherapy protocol. At the end of follow-up, four patients (36.3%) died with a median OS of 15 months. Five patients (45%) survived with a median PFS of 13 months. Two patients were still receiving chemotherapy.

**Conclusion:**

Given the effect of *MYCN* amplification on poor outcome in NB, novel treatments targeting *MYCN* should be developed for patients with NB.

## 1 Introduction

Neuroblastoma (NB), which is an embryonal malignancy, can develop anywhere in the sympathetic nervous system, especially in the abdomen.^[1]^ Its incidence is increasing worldwide. Patients with stage I to III and IVS disease according to the International Neuroblastoma Staging System (INSS) present an excellent outcome.^[2–4]^ However, the prognosis of children with stage IV disease is very poor, with a 5-year event-free survival (EFS) of <50%, even if myeloablative chemotherapy, radiotherapy, immunotherapy, and aggressive surgery are given.^[4–6]^*MYCN* amplification has been considered to be highly correlated with rapid disease progression and poor outcomes^[7]^; however, the potential molecular biological relationship between clinical features and *MYCN* amplification should be further explored.

This case series study was approved by the Ethics Committee of Children's Hospital of Shanghai and based on the clinical data of children with *MYCN*-amplified NB from a single centre. According to known molecular biology results of *MYCN* amplification, we analyzed the relationship between amplification, clinical characteristics, and adverse prognosis to inquire about the possibility of *MYCN* as a therapeutic target.

## 2 Methods

Consecutive child patients (6 males and 5 females) were seen at Shanghai Children's Hospital between August 2017 and September 2019. The diagnosis and staging of NB met the criterion.^[2]^ The patients were examined by fluorescence in situ hybridization (FISH) using a two-color molecular probe for *MYCN* amplifications (ThermoFisher) in tumor mass or bone marrow tissue to determine whether *MYCN* was amplified. The presence of more than four copies of *MYCN* was regarded as amplification. Patients with *MYCN* amplification were included in the study.

The tumor volume of the primary site and the longest dimension were tested at diagnosis and after four cycles of induction chemotherapy based on contrast-enhanced CT. Serum neuron-specific enolase (NSE) levels at diagnosis and after four cycles of induction chemotherapy were calculated. Vanillylmandelic acid (VMA) was tested in 24-h urine collection. All patients were treated according to protocols for NB depending on the children's age and stage and biological features of the disease.^[8,9]^ High-risk patients received four cycles of neoadjuvant chemotherapy (two cycles of cyclophosphamide + topotecan, followed by cisplatin + etoposide and cyclophosphamide + doxorubicin + vindesine + mesna within a 4-week interval). Radical tumor resection was usually performed, followed by tandem high-dose chemotherapy and autologous stem cell transplantation (HDCT/auto-SCT), local radiotherapy and differentiation therapy with 13-cis-retinoic acid. All the treatment procedures were carried out by the multi-disciplinary team (MDT) members, which included experienced oncologists, surgeons, and interventional radiologists. Regular evaluation was performed every 2 months in our department from the end of local radiotherapy. Progressive disease (PD) was defined as any new lesion or an increase in any measurable lesion by >25%.

## 3 Results

Table 1 lists the clinical characteristics of the patients. The age at diagnosis ranged from 6 to 52 months, with a median of 24 months. Before the initial treatment, four cases were diagnosed by histopathology of the tumor, and 7 cases were diagnosed by bone marrow examinations, which included morphology, flow cytometry, and histopathology. Of the 11 patients with *MYCN* amplification, nine were in stage IV (81.8%), one was in stage III (9.1%), and one was in stage I (9.1%). Primary tumors were all located in the abdomen, including the adrenal glands in 10 patients (90.9%) and the paravertebral ganglia in one patient (9.1%). The size of the primary tumors was more than 500 cm^3^, with the longest dimension being over 9 cm in 8 of the 11 patients (72.7%). The most common site of metastasis was bone marrow (72.7%), followed by bone (63.6%) and liver (54.5%). Almost all patients had a higher serum level of NSE than the normal range. NSE was <370 ng/mL in only one patient who had stage I disease, whose primary tumor had a diameter of <5 cm. Eight patients underwent testing for VMA in a 24-h urine sample, of whom 7 had normal levels and 1 had an elevated level. All primary tumors were examined by pathology after 4 to 6 courses of chemotherapy (10/11, 90.9%) or before chemotherapy (1/11). Pathological findings of the primary tumors included poorly differentiated and undifferentiated NB cells in nine cases (81.8%). Because a small number of tumor cells remained, the degree of differentiation could not be judged in the other 2 patients.

**Table 1:**
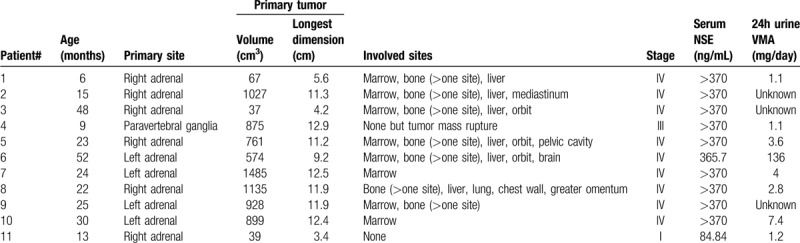
Characteristics of the patients at diagnosis.

The treatment details are shown in Table 2. Eight of 10 (80%) patients who received chemotherapy before resection of the primary tumor showed a good response, with primary tumors reduced by 60% to 99% and serum NSE levels reduced by more than 80% after 4 cycles of chemotherapy. One patient (#7) who did not respond to chemotherapy underwent a second tumor biopsy, and chemotherapy with drugs to which the patient's tumor was sensitive was chosen according to a chemo-drug experiment on biopsy tissues in mice; the patient had a partial response to the second chemotherapy. Except for two patients (#3 and #7), the other patients all underwent radical resection of the primary tumor. During treatment, nonhematologic and hematologic toxicities occurred in almost all patients, but no toxicity-related deaths occurred.

**Table 2:**
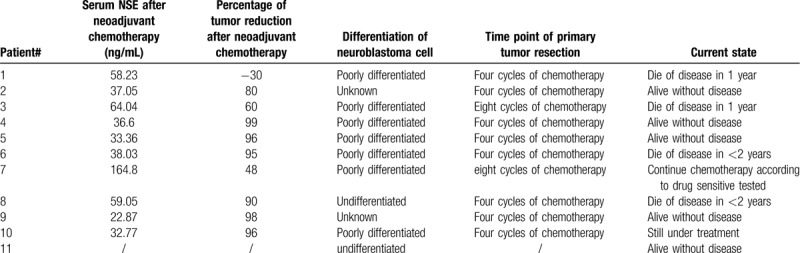
Treatment outcomes according to MYCN amplification.

At the end of follow-up, four patients (#1, #3, #6, and #8; 36.3%) experienced PD and died 1 or 2 years after diagnosis with a median overall survival of 15 months; three of these patients died of lethal brain metastasis. Five patients (45%) survived with a median progression-free survival time of 13 months. Two patients were still under chemotherapy.

## 4 Discussion

Stage IV NB has a poor outcome. It has been a long-held assumption that treatments for *MYCN*-amplified NB need to be more intensive and effective. This concept has provided details on the clinical characteristics and therapeutic responses to current therapeutic strategies of children with *MYCN-*amplified NB in China. Our data from these patients showed that all the children were <5 years old, and the primary tumors were located in the retroperitoneum, which are similar to the results in the literature.^[10]^ We further found that tumors were far more common in the adrenal glands than in the ganglia in the abdomen. NSE and VMA, which are specific biomarkers for the NB response arising from the secretory functions of tumor cells, were present at different levels, with most patients having higher NSE and lower VAM levels than the respective normal ranges, in accordance with the findings of *Lee JW*, who showed that NB with *MYCN* amplification was related to a higher NSE, lower VMA, and poorer differentiation.^[10]^ VMA is probably secreted from more mature tumor cells.^[11]^ No ganglioma cells were found, and most cells were poorly differentiated or undifferentiated in pathological sections of all the children's tumors. These results can explain the low levels of VMA and indicate that NB cells with *MYCN* amplification are extremely immature. At the time of diagnosis, extensive metastasis had already appeared, especially metastasis to bone marrow.

All patients, except for one with stage I disease, accepted neoadjuvant chemotherapy protocols based on age, *MYCN* amplification status, image-defined risk factors (IDRFs) and INSS stage, and the chemotherapy drugs included cyclophosphamide, ifosfamide, cisplatin, carboplatin, and adriamycin. One child who was not sensitive to the indicated chemotherapy regimen adopted adjusted schemes after a drug sensitivity test. He was eligible for removal of the primary tumor after four cycles of modified chemotherapy. Therefore, for children who do not respond to initial chemotherapy, a drug sensitivity test of tumor tissue is a feasible choice for identifying strategies to which the tumor will be sensitive. In our study, most patients responded well to therapy, with an obvious decrease in NSE and tumor volume. The tumor cells in the bone marrow were usually cleared out after two cycles of chemotherapy, with negative results on morphology and flow cytometry analyses. However, disease progression rapidly occurred in the form of recurrence at metastatic sites after all courses of treatment in four patients, with a median OS of 15 months after diagnosis. Although five patients are still alive, only the survival period of the patient with stage I disease exceeded 15 months. This result shows that the currently used therapeutic strategy, comprising chemotherapy, HDCT/auto-SCT, surgery, and radiotherapy, can effectively inhibit the primary tumor but does not control metastatic cells well. Perhaps more intensive or novel treatments should be explored. In a prospective study, high-dose combined chemotherapy did not improve the prognosis of stage IV patients with *MYCN* amplification.^[12]^ The efficacy of anti-GD2 antibody in relapsed or refractory patients remains under investigation.^[13]^ Although immunotherapy with maintenance therapy was found to increase 2-year survival in patients with recurrent or refractory NB,^[14]^ it was not analyzed in patients with *MYCN* amplification, and the 5-year and longer survival values are still unknown. Patients with *MYCN* amplification often have recurrent or refractory disease. Thus, a more effective treatment modality should target *MYCN*.

Approaches combating *MYCN* amplification may need to be considered. *MYCN* is a member of the *MYC* oncogene family. It is a transcription factor that can regulate the expression of many target genes and participates in cell growth, apoptosis, tumor invasion, and metastasis. In normal cells, the role of *MYCN,* which is located on the terminal end of the short arm of chromosome 2, is to shorten the cycle of cell growth, promote proliferation, and inhibit cell differentiation. Since *MYCN* promotes the proliferation of cells and inhibits differentiation, the *MYCN-*amplified cells are often undifferentiated or poorly differentiated,^[15,16]^ which is consistent with the poor differentiation of the tumor cells in our clinical patients. In differentiated neurons, the expression of *MYCN* is downregulated. *MYCN*-driven transgenic mouse models and primary neural crest cells successfully recapitulate human disease,^[17,18]^ demonstrating the key role of *MYCN* in NB tumorigenesis. Furthermore, *MYCN* is related to tumor drug resistance. A large prospective study showed that *MYCN* amplification was associated with higher expression of multiple resistance protein 1 (MRP1).^[19]^ In an in vitro study, high expression of *MYCN* increased the expression of MRP1, resulting in failure of chemotherapy due to low intracellular drug concentration.^[20]^ Therefore, inhibiting the expression of *MYCN* at the transcriptional or translational level may improve antitumor efficacy. A series of proteasomes have been identified,^[21]^ which can be synthetic lethal targets in this type of NB. In our observational study, the size of the cohort was relatively small, and the follow-up duration was short, which might limit the value of the analysis in the study. However, based on these findings, *MYCN*-amplified NB should be considered carefully, and more novel treatment modalities should be considered by pediatric oncologists.

## 5 Conclusions

*MYCN* amplification is associated with poor prognosis although patients have an encouraging initial response to currently used chemotherapies. New treatment modalities need to be explored to improve outcomes, especially those targeting *MYCN*.

## Author contributions

All the Authors made substantial contributions to conception and design of the study. Can Huang and Shayi Jiang conceived the study and wrote the paper; Jingwei Yang, Xuelian Liao, Yanhua Li, Shanshan Li performed literature search and reviewed the paper.
